# Supplementary material for: G-protein coupled receptor 19 (GPR19) knockout mice display sex-dependent metabolic dysfunction
Source: Sci Rep. 2023 Apr 15;13:6134. doi: 10.1038/s41598-023-33308-7 (PMC10105709; doi:10.1038/s41598-023-33308-7)
Supplement: Supplementary file 1 — Supplementary Information 1. [file 41598_2023_33308_MOESM1_ESM.pdf]

**Supplemental Table 1**

| | | | | | |
|---|---|---|---|---|---|
| Table Analyzed | Feeding avg per mouse Males LFD HFD | | | | |
| Two-way ANOVA | Ordinary | | | | |
| Alpha | 0.05 | | | | |
| Source of Variation | % of total variation | P value | P value summary | Significant? | |
| Interaction | 2.810 | 0.2996 | ns | No | |
| Row Factor | 16.48 | <0.0001 | **** | Yes | |
| Column Factor | 48.63 | <0.0001 | **** | Yes | |
| ANOVA table | SS (Type III) | DF | MS | F (DFn, DFd) | P value |
| Interaction | 5.520 | 6 | 0.9200 | F (6, 84) = 1.229 | P=0.2996 |
| Row Factor | 32.37 | 2 | 16.18 | F (2, 84) = 21.63 | P<0.0001 |
| Column Factor | 95.51 | 3 | 31.84 | F (3, 84) = 42.54 | P<0.0001 |
| Residual | 62.86 | 84 | 0.7484 | | |
| Data summary | | | | | |
| Number of columns (Column Factor) | 4 | | | | |
| Number of rows (Row Factor) | 3 | | | | |
| Number of values | 96 | | | | |

| | | | | | |
|---|---|---|---|---|---|
| Table Analyzed | EE avg per mouse Males LFD HFD | | | | |
| Two-way ANOVA | Ordinary | | | | |
| Alpha | 0.05 | | | | |
| Source of Variation | % of total variation | P value | P value summary | Significant? | |
| Interaction | 0.2190 | 0.9973 | ns | No | |
| Row Factor | 34.52 | <0.0001 | **** | Yes | |
| Column Factor | 30.59 | <0.0001 | **** | Yes | |
| ANOVA table | SS (Type III) | DF | MS | F (DFn, DFd) | P value |
| Interaction | 0.001315 | 6 | 0.0002192 | F (6, 84) = 0.08885 | P=0.9973 |
| Row Factor | 0.2073 | 2 | 0.1037 | F (2, 84) = 42.01 | P<0.0001 |
| Column Factor | 0.1837 | 3 | 0.06124 | F (3, 84) = 24.82 | P<0.0001 |
| Residual | 0.2073 | 84 | 0.002467 | | |
| Data summary | | | | | |
| Number of columns (Column Factor) | 4 | | | | |
| Number of rows (Row Factor) | 3 | | | | |

| | |
|---|---|
| Number of values | 96 |

| | | | | | |
|---|---|---|---|---|---|
| Table Analyzed | Total activity per mouse Males LFD HFD | | | | |
| Two-way ANOVA | Ordinary | | | | |
| Alpha | 0.05 | | | | |
| Source of Variation | % of total variation | P value | P value summary | Significant? | |
| Interaction | 0.7613 | 0.9678 | ns | No | |
| Row Factor | 37.08 | <0.0001 | **** | Yes | |
| Column Factor | 13.07 | 0.0001 | *** | Yes | |
| ANOVA table | SS (Type III) | DF | MS | F (DFn, DFd) | P value |
| Interaction | 4650 | 6 | 775.0 | F (6, 84) = 0.2245 | P=0.9678 |
| Row Factor | 226494 | 2 | 113247 | F (2, 84) = 32.80 | P<0.0001 |
| Column Factor | 79811 | 3 | 26604 | F (3, 84) = 7.705 | P=0.0001 |
| Residual | 290019 | 84 | 3453 | | |
| Data summary | | | | | |
| Number of columns (Column Factor) | 4 | | | | |
| Number of rows (Row Factor) | 3 | | | | |
| Number of values | 96 | | | | |

| | | | | | |
|---|---|---|---|---|---|
| Table Analyzed | RQ avg per mouse Males LFD HFD | | | | |
| Two-way ANOVA | Ordinary | | | | |
| Alpha | 0.05 | | | | |
| Source of Variation | % of total variation | P value | P value summary | Significant? | |
| Interaction | 9.582 | 0.0004 | *** | Yes | |
| Row Factor | 8.542 | <0.0001 | **** | Yes | |
| Column Factor | 53.87 | <0.0001 | **** | Yes | |
| ANOVA table | SS (Type III) | DF | MS | F (DFn, DFd) | P value |
| Interaction | 0.01221 | 6 | 0.002035 | F (6, 84) = 4.702 | P=0.0004 |
| Row Factor | 0.01088 | 2 | 0.005442 | F (2, 84) = 12.58 | P<0.0001 |
| Column Factor | 0.06865 | 3 | 0.02288 | F (3, 84) = 52.88 | P<0.0001 |
| Residual | 0.03635 | 84 | 0.0004328 | | |
| Data summary | | | | | |
| Number of columns (Column Factor) | 4 | | | | |
| Number of rows (Row Factor) | 3 | | | | |
| Number of values | 96 | | | | |

| | | | | | |
|---|---|---|---|---|---|
| Table Analyzed | BW and Comp Males LFD HFD | | | | |
| Two-way ANOVA | Ordinary | | | | |
| Alpha | 0.05 | | | | |
| Source of Variation | % of total variation | P value | P value summary | Significant? | |
| Interaction | 3.785 | <0.0001 | **** | Yes | |
| Row Factor | 75.50 | <0.0001 | **** | Yes | |
| Column Factor | 16.72 | <0.0001 | **** | Yes | |
| ANOVA table | SS (Type III) | DF | MS | F (DFn, DFd) | P value |
| Interaction | 601.1 | 6 | 100.2 | F (6, 84) = 17.47 | P<0.0001 |
| Row Factor | 11989 | 2 | 5995 | F (2, 84) = 1045 | P<0.0001 |
| Column Factor | 2656 | 3 | 885.2 | F (3, 84) = 154.3 | P<0.0001 |
| Residual | 481.8 | 84 | 5.736 | | |
| Data summary | | | | | |
| Number of columns (Column Factor) | 4 | | | | |
| Number of rows (Row Factor) | 3 | | | | |
| Number of values | 96 | | | | |

| | | | | | |
|---|---|---|---|---|---|
| Table Analyzed | EE avg per mouse Females LFD HFD | | | | |
| Two-way ANOVA | Ordinary | | | | |
| Alpha | 0.05 | | | | |
| Source of Variation | % of total variation | P value | P value summary | Significant? | |
| Interaction | 1.428 | 0.2237 | ns | No | |
| Row Factor | 56.42 | <0.0001 | **** | Yes | |
| Column Factor | 25.44 | <0.0001 | **** | Yes | |
| ANOVA table | SS (Type III) | DF | MS | F (DFn, DFd) | P value |
| Interaction | 0.005325 | 6 | 0.0008874 | F (6, 84) = 1.401 | P=0.2237 |
| Row Factor | 0.2104 | 2 | 0.1052 | F (2, 84) = 166.1 | P<0.0001 |
| Column Factor | 0.09486 | 3 | 0.03162 | F (3, 84) = 49.93 | P<0.0001 |
| Residual | 0.05320 | 84 | 0.0006333 | | |
| Data summary | | | | | |
| Number of columns (Column Factor) | 4 | | | | |
| Number of rows (Row Factor) | 3 | | | | |

| | |
|---|---|
| Number of values | 96 |

| | | | | | |
|---|---|---|---|---|---|
| Table Analyzed | Feeding avg per mouse Females LFD HFD | | | | |
| Two-way ANOVA | Ordinary | | | | |
| Alpha | 0.05 | | | | |
| Source of Variation | % of total variation | P value | P value summary | Significant? | |
| Interaction | 2.445 | 0.4214 | ns | No | |
| Row Factor | 13.63 | <0.0001 | **** | Yes | |
| Column Factor | 50.01 | <0.0001 | **** | Yes | |
| ANOVA table | SS (Type III) | DF | MS | F (DFn, DFd) | P value |
| Interaction | 4.155 | 6 | 0.6925 | F (6, 84) = 1.015 | P=0.4214 |
| Row Factor | 23.17 | 2 | 11.58 | F (2, 84) = 16.97 | P<0.0001 |
| Column Factor | 85.00 | 3 | 28.33 | F (3, 84) = 41.52 | P<0.0001 |
| Residual | 57.33 | 84 | 0.6825 | | |
| Data summary | | | | | |
| Number of columns (Column Factor) | 4 | | | | |
| Number of rows (Row Factor) | 3 | | | | |
| Number of values | 96 | | | | |

| | | | | | |
|---|---|---|---|---|---|
| Table Analyzed | Total activity per mouse Females LFD HFD | | | | |
| Two-way ANOVA | Ordinary | | | | |
| Alpha | 0.05 | | | | |
| Source of Variation | % of total variation | P value | P value summary | Significant? | |
| Interaction | 1.925 | 0.6285 | ns | No | |
| Row Factor | 31.92 | <0.0001 | **** | Yes | |
| Column Factor | 27.00 | <0.0001 | **** | Yes | |
| ANOVA table | SS (Type III) | DF | MS | F (DFn, DFd) | P value |
| Interaction | 17756 | 6 | 2959 | F (6, 84) = 0.7278 | P=0.6285 |
| Row Factor | 294370 | 2 | 147185 | F (2, 84) = 36.20 | P<0.0001 |
| Column Factor | 248980 | 3 | 82993 | F (3, 84) = 20.41 | P<0.0001 |
| Residual | 341549 | 84 | 4066 | | |
| Data summary | | | | | |
| Number of columns (Column Factor) | 4 | | | | |
| Number of rows (Row Factor) | 3 | | | | |
| Number of values | 96 | | | | |

| | | | | | |
|---|---|---|---|---|---|
| Table Analyzed | RQ avg oer mouse Females LFD HFD | | | | |
| Two-way ANOVA | Ordinary | | | | |
| Alpha | 0.05 | | | | |
| Source of Variation | % of total variation | P value | P value summary | Significant? | |
| Interaction | 2.557 | 0.7447 | ns | No | |
| Row Factor | 3.759 | 0.0832 | ns | No | |
| Column Factor | 32.35 | <0.0001 | **** | Yes | |
| ANOVA table | SS (Type III) | DF | MS | F (DFn, DFd) | P value |
| Interaction | 0.003088 | 6 | 0.0005146 | F (6, 84) = 0.5807 | P=0.7447 |
| Row Factor | 0.004539 | 2 | 0.002269 | F (2, 84) = 2.561 | P=0.0832 |
| Column Factor | 0.03906 | 3 | 0.01302 | F (3, 84) = 14.69 | P<0.0001 |
| Residual | 0.07444 | 84 | 0.0008861 | | |
| Data summary | | | | | |
| Number of columns (Column Factor) | 4 | | | | |
| Number of rows (Row Factor) | 3 | | | | |
| Number of values | 96 | | | | |

| | | | | | |
|---|---|---|---|---|---|
| Table Analyzed | BW and Comp Females LFD HFD | | | | |
| Two-way ANOVA | Ordinary | | | | |
| Alpha | 0.05 | | | | |
| Source of Variation | % of total variation | P value | P value summary | Significant? | |
| Interaction | 10.41 | <0.0001 | **** | Yes | |
| Row Factor | 53.08 | <0.0001 | **** | Yes | |
| Column Factor | 31.07 | <0.0001 | **** | Yes | |
| ANOVA table | SS (Type III) | DF | MS | F (DFn, DFd) | P value |
| Interaction | 1318 | 6 | 219.6 | F (6, 84) = 30.07 | P<0.0001 |
| Row Factor | 6722 | 2 | 3361 | F (2, 84) = 460.1 | P<0.0001 |
| Column Factor | 3934 | 3 | 1311 | F (3, 84) = 179.6 | P<0.0001 |
| Residual | 613.5 | 84 | 7.304 | | |
| Data summary | | | | | |
| Number of columns (Column Factor) | 4 | | | | |
| Number of rows (Row Factor) | 3 | | | | |
| Number of values | 96 | | | | |
