# Supplementary figures and images for: G-protein coupled receptor 19 (GPR19) knockout mice display sex-dependent metabolic dysfunction
Source: Sci Rep. 2023 Apr 15;13:6134. doi: 10.1038/s41598-023-33308-7 (PMC10105709; doi:10.1038/s41598-023-33308-7)

Supplemental Figure 1

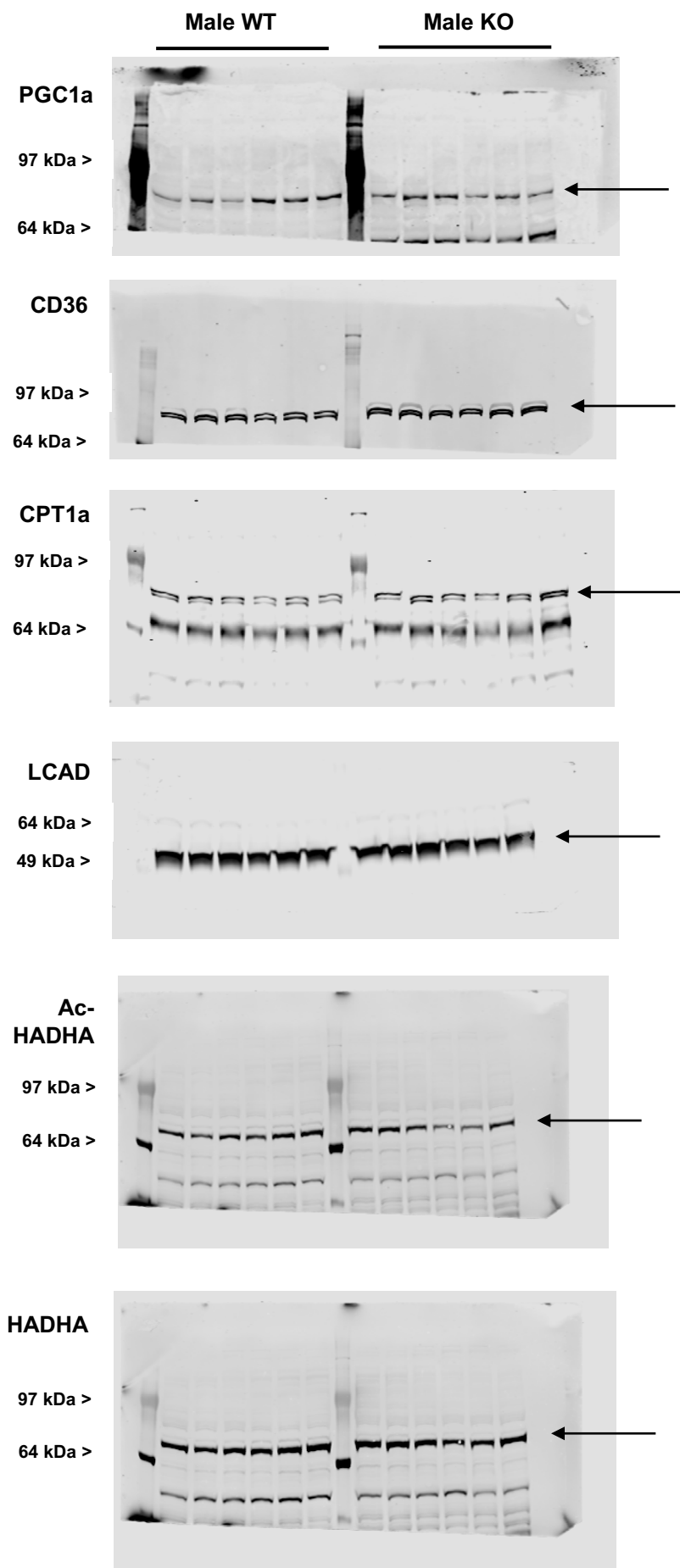

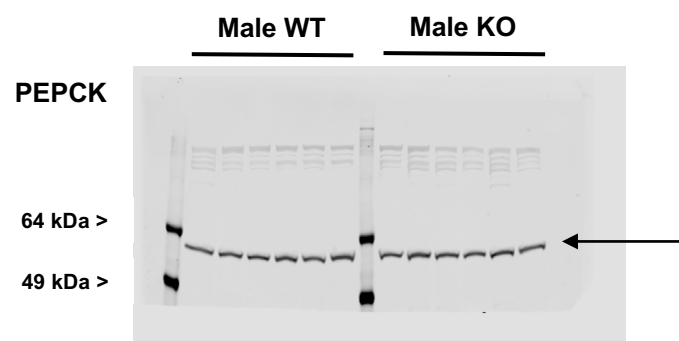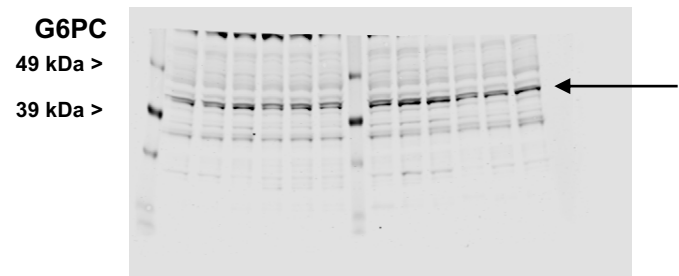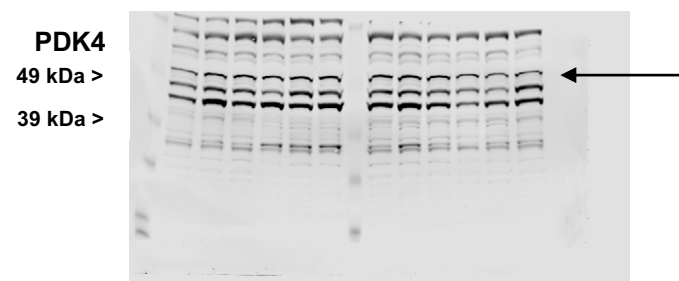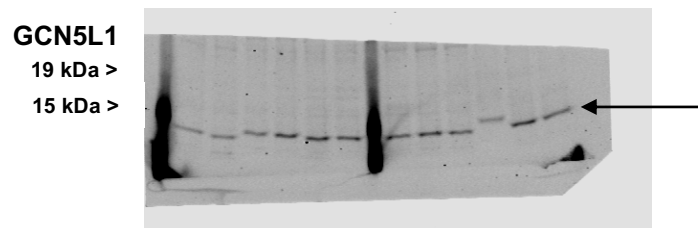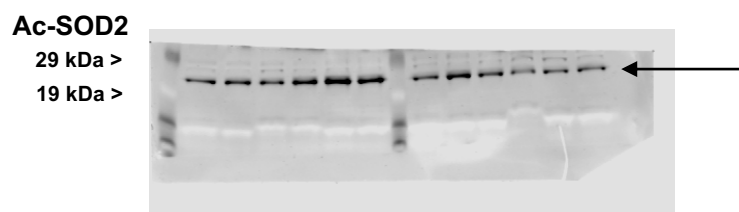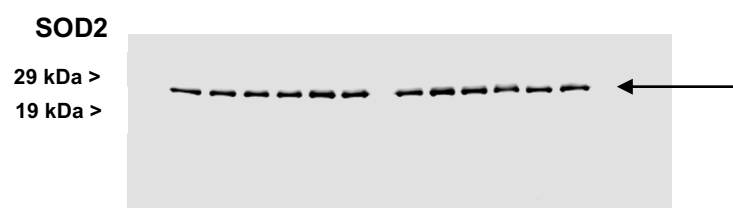

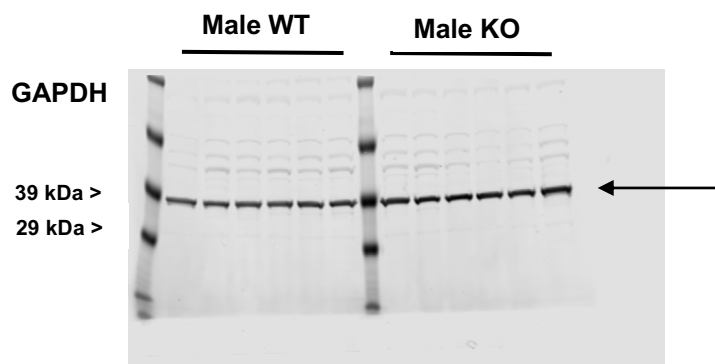

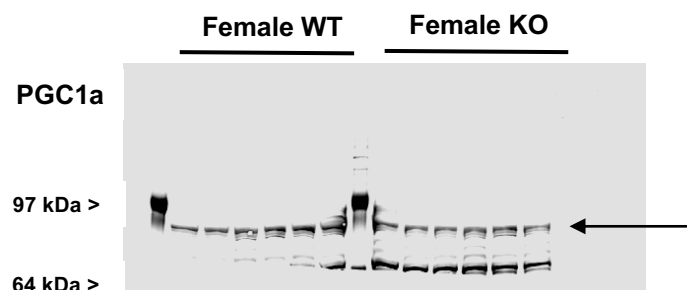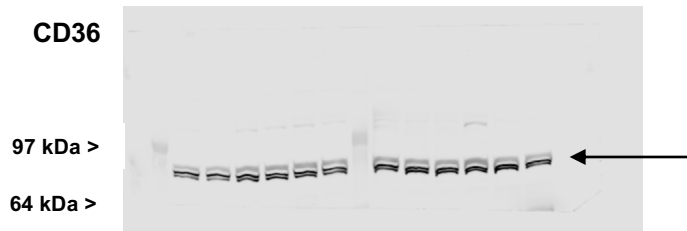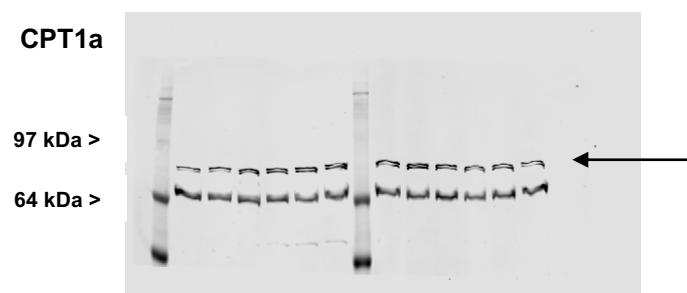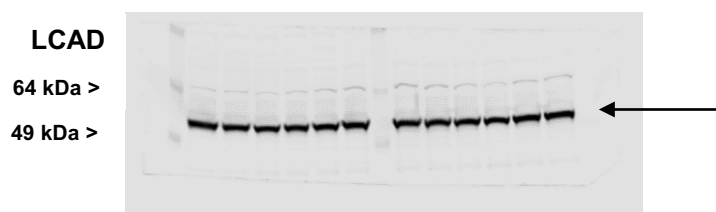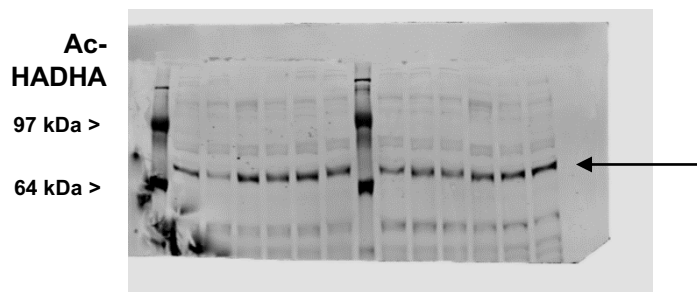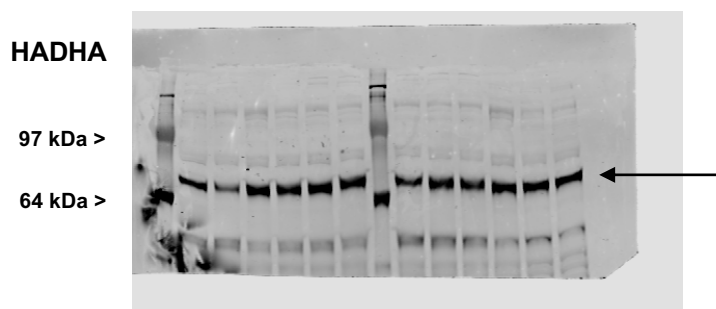

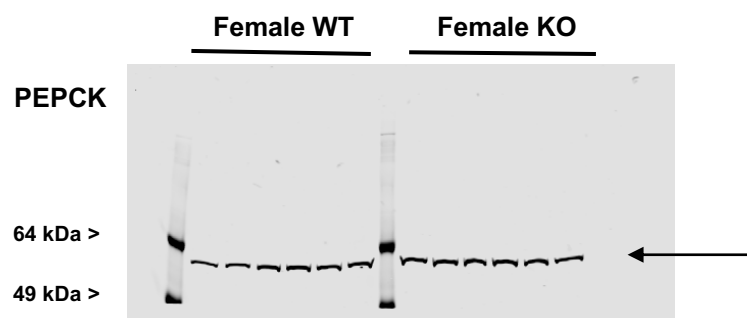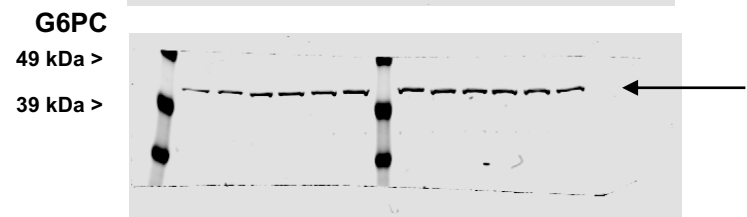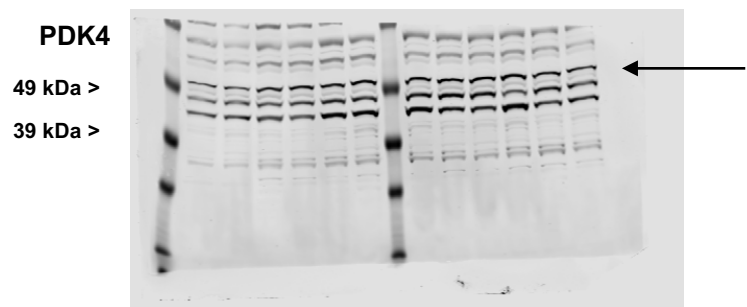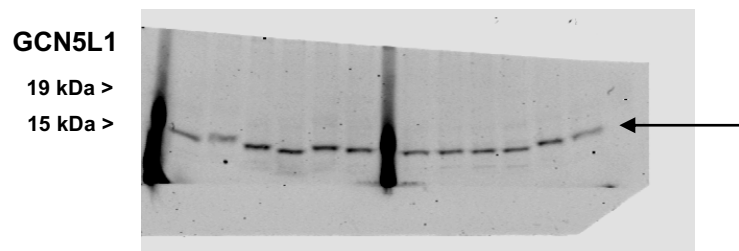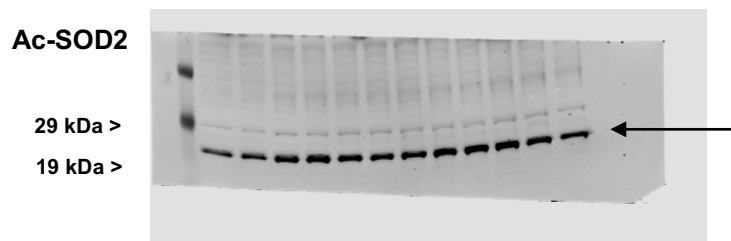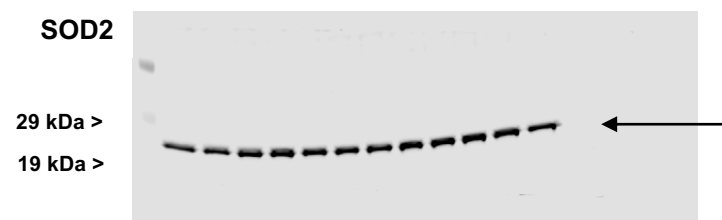

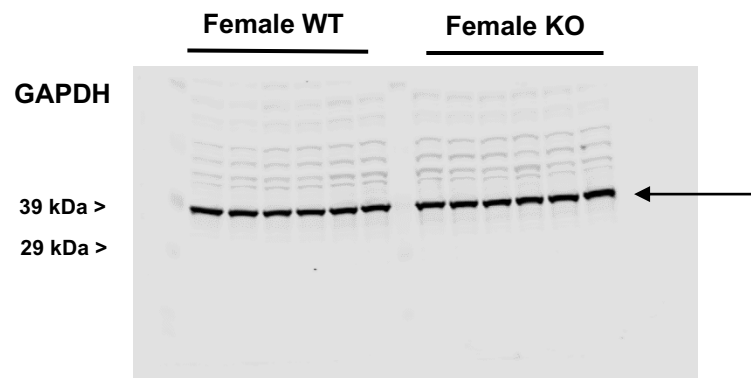

Supplement: Supplementary file 2 — Supplementary Information 2. [file 41598_2023_33308_MOESM2_ESM.pdf]

### Chow GTT

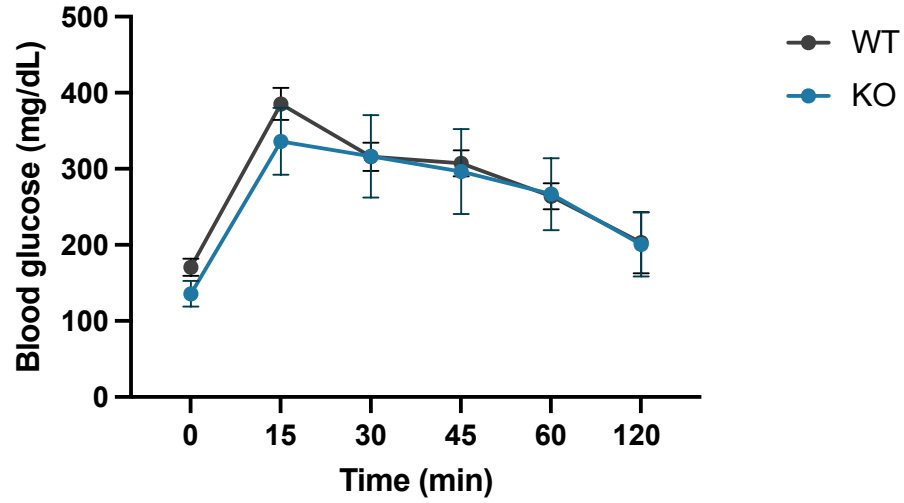

### Insulin Time Course

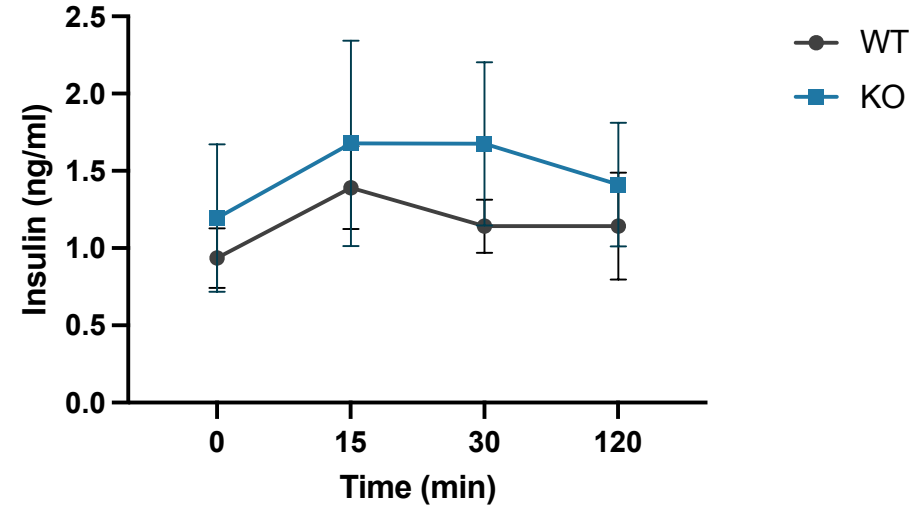

### Glucose AUC

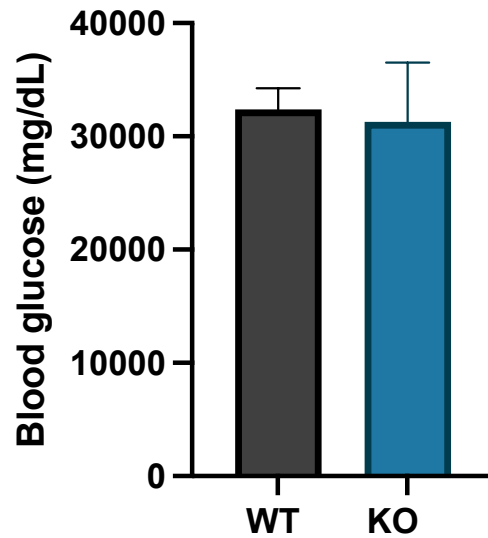

### Body Weight

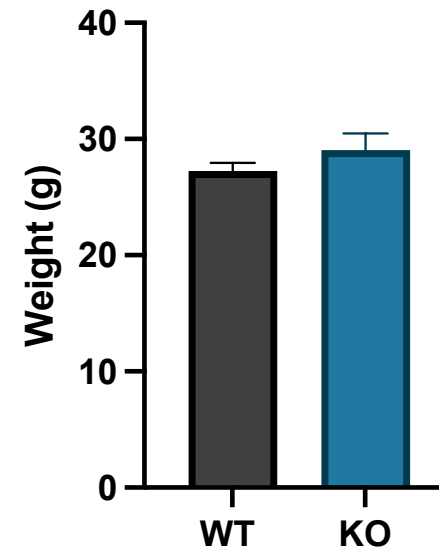

Supplement: Supplementary file 3 — Supplementary Information 3. [file 41598_2023_33308_MOESM3_ESM.pdf]

## Male Basal Insulin

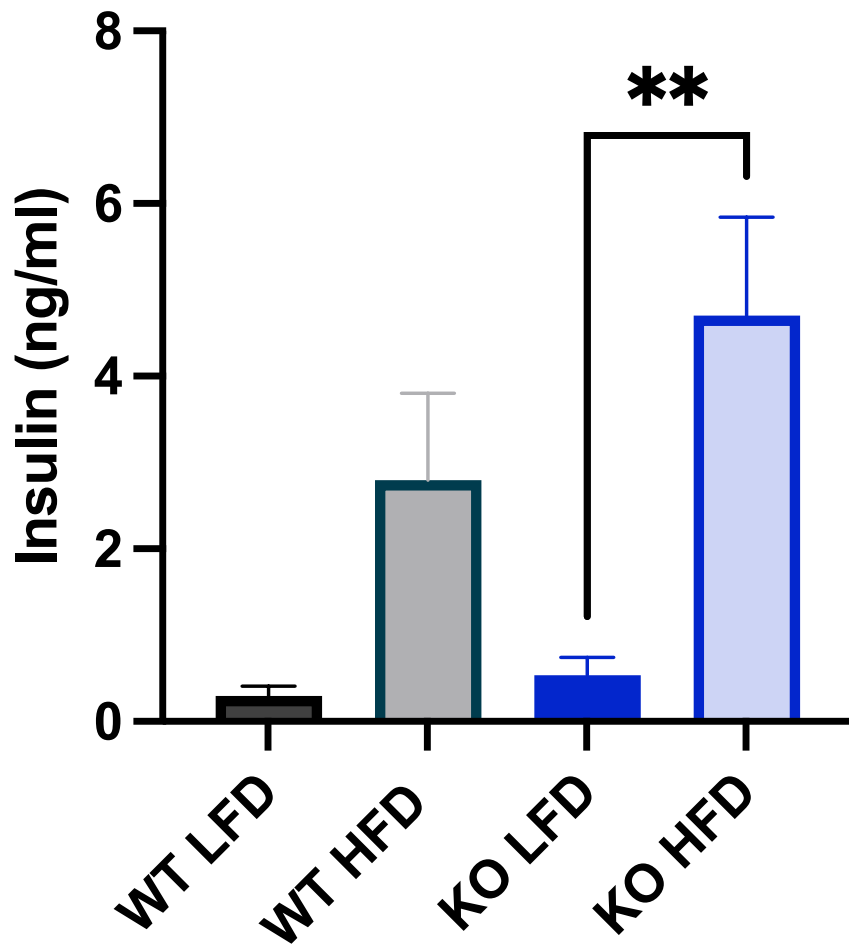

## Female Basal Insulin

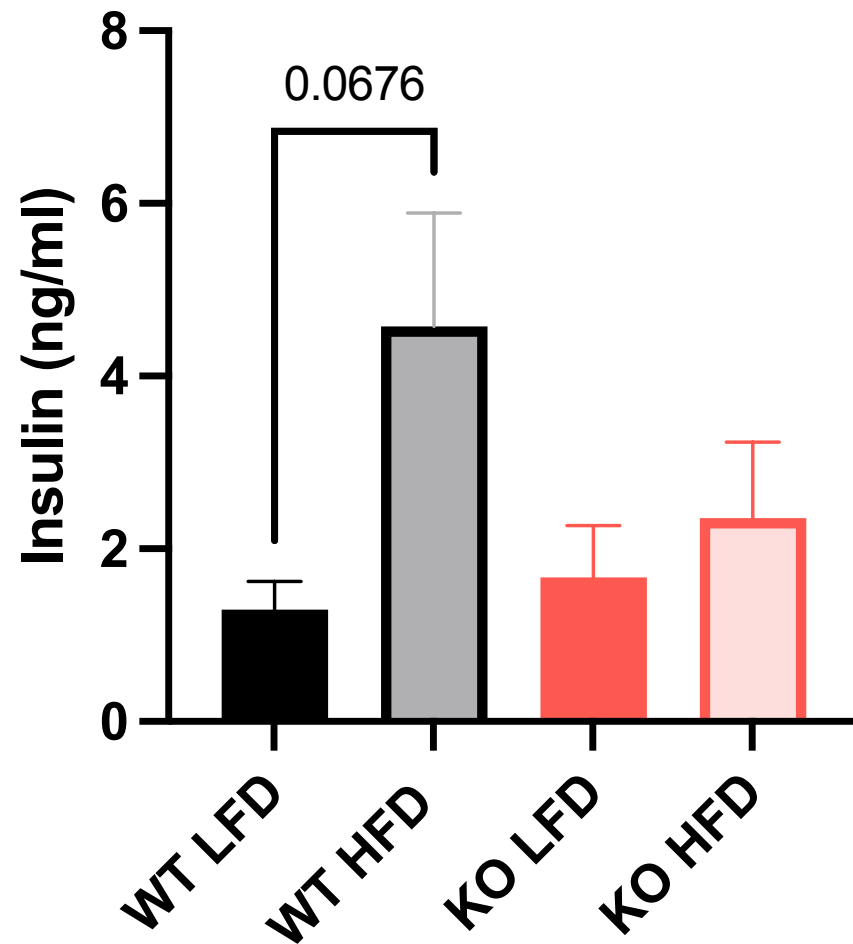

Supplement: Supplementary file 4 — Supplementary Information 4. [file 41598_2023_33308_MOESM4_ESM.pdf]
