# Supplementary material for: G-protein coupled receptor 19 (GPR19) knockout mice display sex-dependent metabolic dysfunction
Source: Sci Rep. 2023 Apr 15;13:6134. doi: 10.1038/s41598-023-33308-7 (PMC10105709; doi:10.1038/s41598-023-33308-7)
Supplement: Supplementary file 5 — Supplementary Information 5. [file 41598_2023_33308_MOESM5_ESM.pdf]

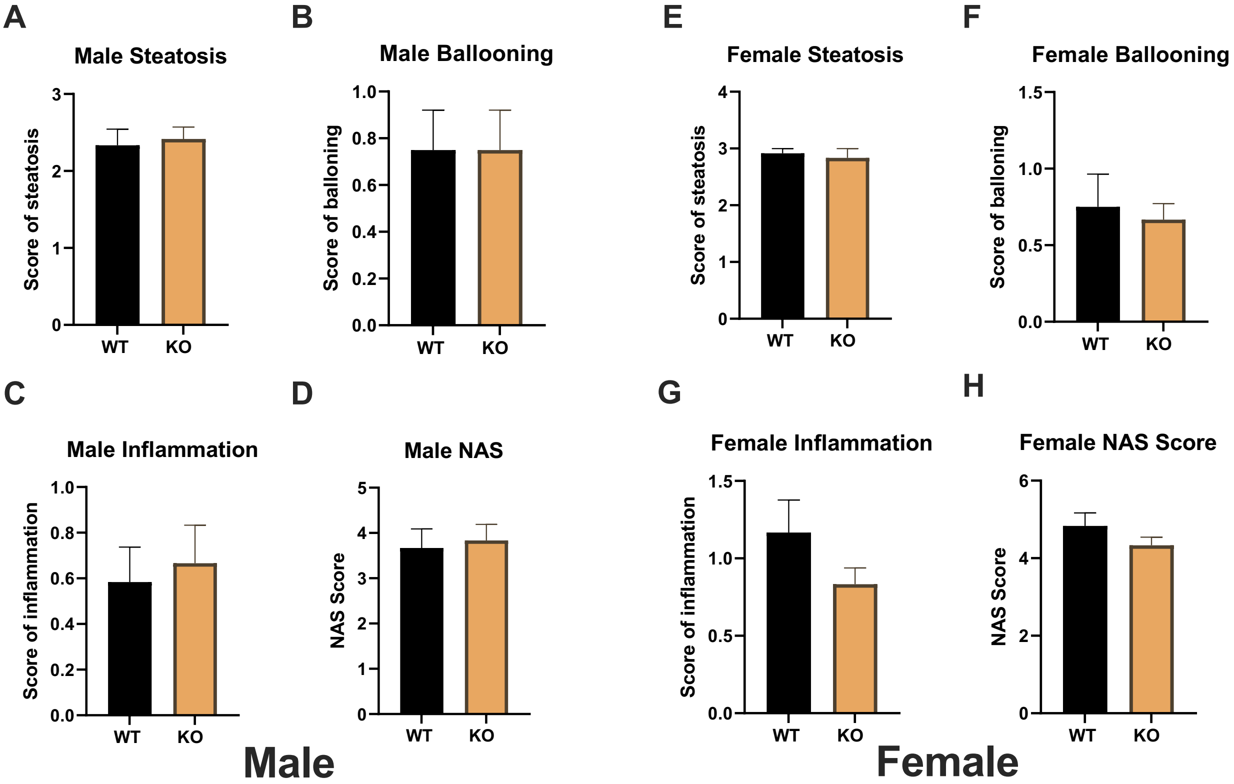
A
Male Steatosis
Score of steatosis
3
2
1
0
WT
KO
B
Male Ballooning
Score of balloning
1.0
0.8
0.6
0.4
0.2
0.0
WT
KO
E
Female Steatosis
Score of steatosis
4
3
2
1
0
WT
KO
F
Female Ballooning
Score of balloning
1.5
1.0
0.5
0.0
WT
KO
C
Male Inflammation
Score of inflammation
1.0
0.8
0.6
0.4
0.2
0.0
WT
KO
D
Male NAS
NAS Score
5
4
3
2
1
0
WT
KO
G
Female Inflammation
Score of inflammation
1.5
1.0
0.5
0.0
WT
KO
H
Female NAS Score
NAS Score
6
4
2
0
WT
KO
Male
Female
